# Supplementary material for: Modified N-linked glycosylation status predicts trafficking defective human Piezo1 channel mutations
Source: Commun Biol. 2021 Sep 6;4:1038. doi: 10.1038/s42003-021-02528-w (PMC8421374; doi:10.1038/s42003-021-02528-w)
Supplement: Supplementary file 1 — Supplementary Information [file 42003_2021_2528_MOESM1_ESM.pdf]

Supplementary information

Modified N-linked glycosylation status predicts trafficking defective human Piezo1 channel mutations

Jinyuan Vero Li[1], Chai-Ann Ng[1,2], Delfine Cheng[1,2], Zijing Zhou[1], Mingxi Yao[3], Yang Guo[1,2], Ze-Yan Yu[1,2], Yogambha Ramaswamy[4], Lining Arnold Ju[4], Philip W Kuchel[5], Michael P Feneley[1,2,6], Diane Fatkin[1,2] & Charles D Cox[1,2#]

[1]Molecular Cardiology and Biophysics Division, Victor Chang Cardiac Research Institute, Sydney, Australia. [2] St Vincent's Clinical School, Faculty of Medicine, University of New South Wales, Sydney, Australia. [3]Mechanobiology Institute, National University of Singapore, Singapore. [4]School of Biomedical Engineering, Faculty of Engineering, The University of Sydney, Camperdown, New South Wales, Australia. [5]School of Life and Environmental Sciences, University of Sydney, Sydney, New South Wales, Australia. [6]Department of Cardiology, St Vincent's Hospital, Sydney, Australia.

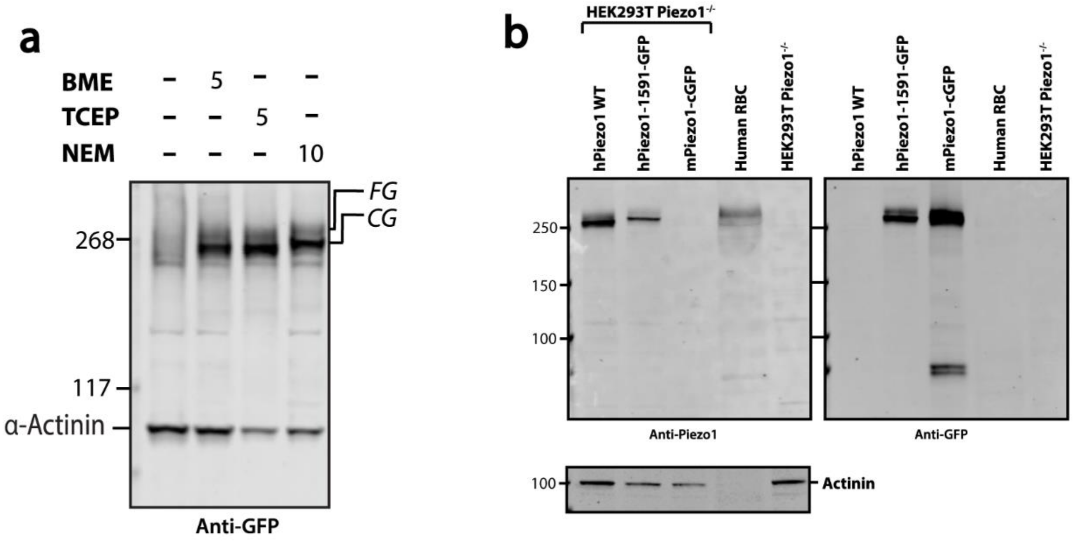

Supplementary Figure 1. Primary monoclonal Piezo1 antibody (NBP2-75617 Novus Biologicals) reproducibly recognizes human Piezo1 but fails to recognize mouse Piezo1. (a) A representative western blot of Piezo1-1591-GFP fusion lysate with no reducing agent present in the lysis buffer compared with the addition of 5 mM BME, 5 mM TCEP and 10 mM N-ethylmaleimide expressed in Piezo1$^{-/-}$ HEK293T. Notice the doublet on the Western blot in the presence of a reducing agent or alkylating agent. (b) Left panel -Representative Western blot using Novus primary monoclonal (Cat# NBP2-75617, Novus Biologicals) anti-Piezo1 antibody with samples of untagged human Piezo1, Piezo1-1591-GFP fusion and mouse Piezo1-C-terminal-GFP expressed in HEK293T Piezo1$^{-/-}$ compared to human red blood cell lysate and HEK293T Piezo1$^{-/-}$ lysate as a negative control. Right panel- Same blot as that shown in left panel probed with an anti-GFP antibody.

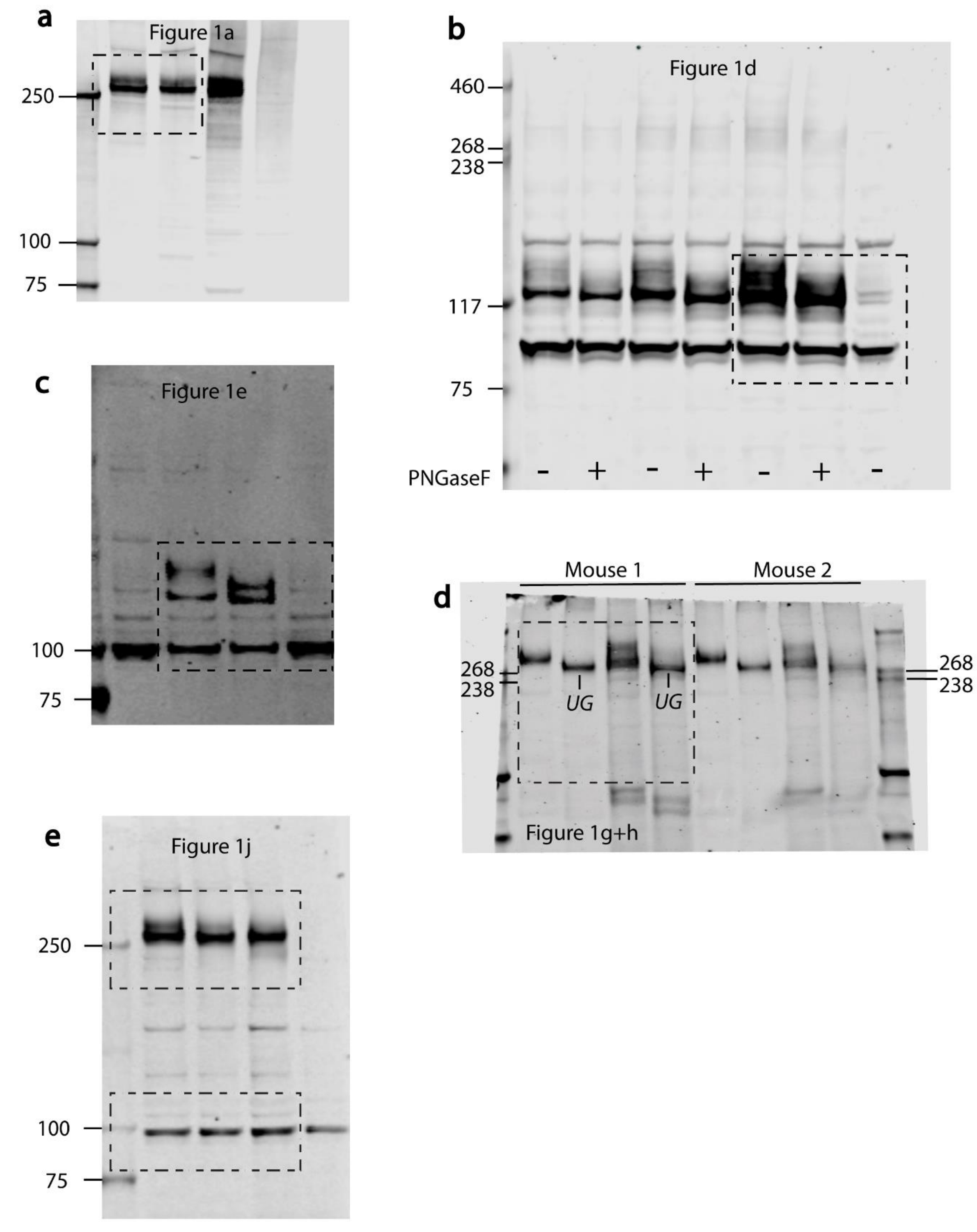

Supplementary Figure 2. Full blots from main text Figure 1. (a) Blot Figure 1a, (b) Blot Figure 1d, (c) Blot Figure 1e, (d) Blot Figure 1g, 1h, (e) Blot Figure 1j.

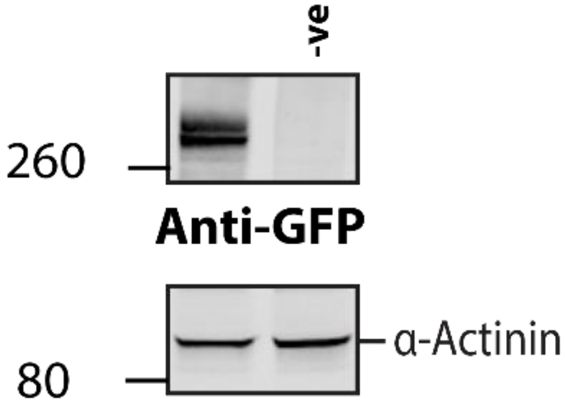

Supplementary Figure 3. Piezo1 expression in Neuro2a also runs as two bands on western blots. Representative western blot of transiently transfected Neuro2a cells with Piezo1-GFP and a vector transfected negative control.

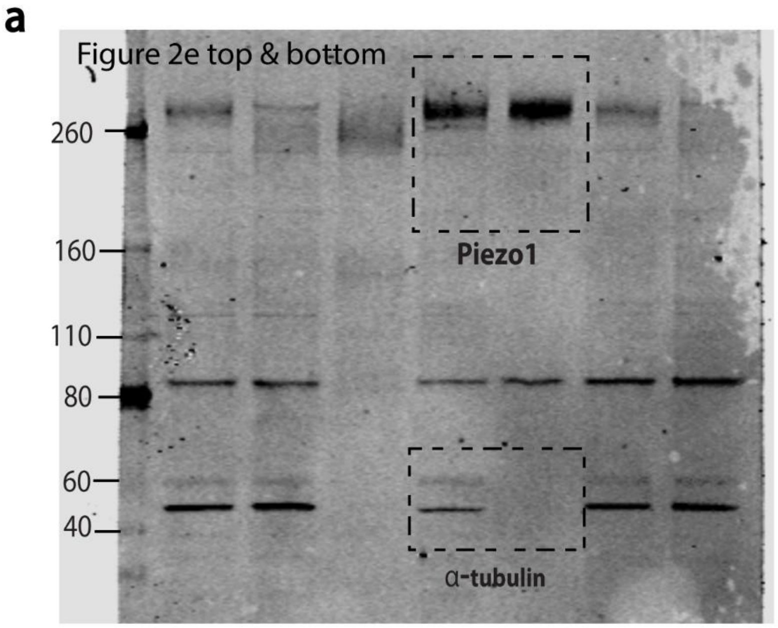

Supplementary Figure 4. Full blot of main text Figure 2. (a) Blot figure 2e. Dashed lines represent cropped areas.

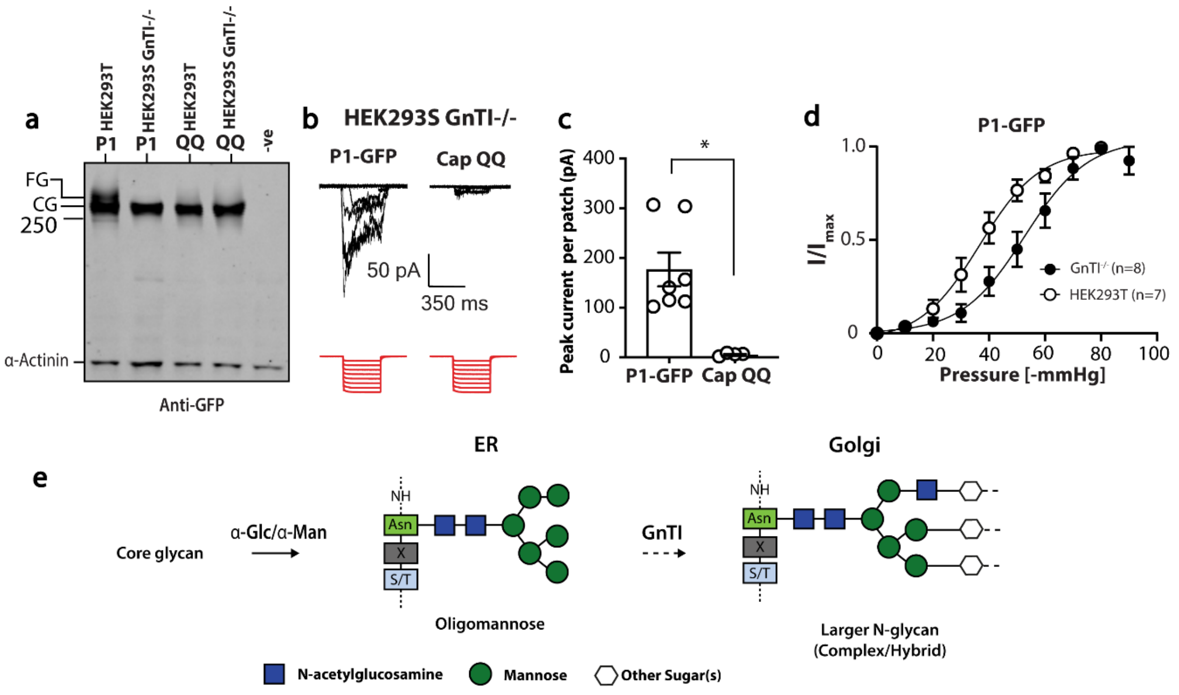

Supplementary Figure 5. Higher order glycosylation does not happen in GnT1-/- HEK293S cells. (a) Representative western blot comparing Piezo1-GFP (P1-GFP), CapQQ mutant expressed in HEK293T Piezo1$^{-/-}$ and HEK293S GnT1$^{-/-}$. (b) Electrophysiological recordings of HEK293S GnT1$^{-/-}$ expressing Piezo1-GFP and CapQQ in the cell-attached configuration in response to negative pressure applied using a high-speed pressure-clamp (red). (c) Quantification of peak current elicited per patch of Piezo1-GFP and CapQQ expressed in HEK293S GnT1$^{-/-}$. (d) Pressure response curve of Piezo1-GFP expressed in Piezo1$^{-/-}$ HEK293T compared to Gnt1-/- HEK293S. (e) A cartoon representing N-acetylglucosaminyl-transferase I (GnT1) enzyme processing of N-glycans from high-mannose to higher molecular weight glycans.

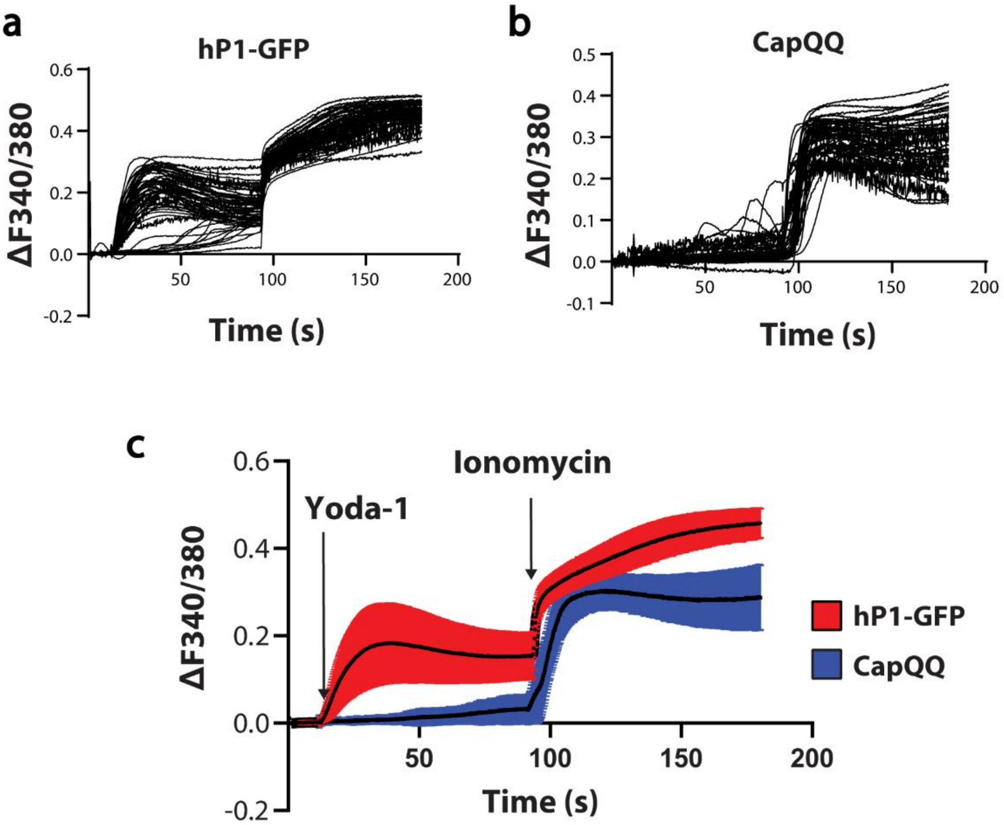

Supplementary Figure 6. Yoda-1 fails to elicit a Ca^{2+} response in HEK293T Piezo1$^{-/-}$ cells transfected with the CapQQ mutant. (a) Individual cell responses to 2 µM Yoda-1 and 5uM ionomycin for HEK293T Piezo1$^{-/-}$ cells transfected with human Piezo1 (hP1-GFP). (b) Individual cell responses to 2 µM Yoda-1 and 5 µM ionomycin for HEK293T Piezo1$^{-/-}$ cells transfected with CapQQ. (c) Comparison between the response of Piezo1 and the CapQQ mutant to Yoda-1 stimulation (2 µM). Data shows n=70 cells from three independent experiments - mean ± SD.

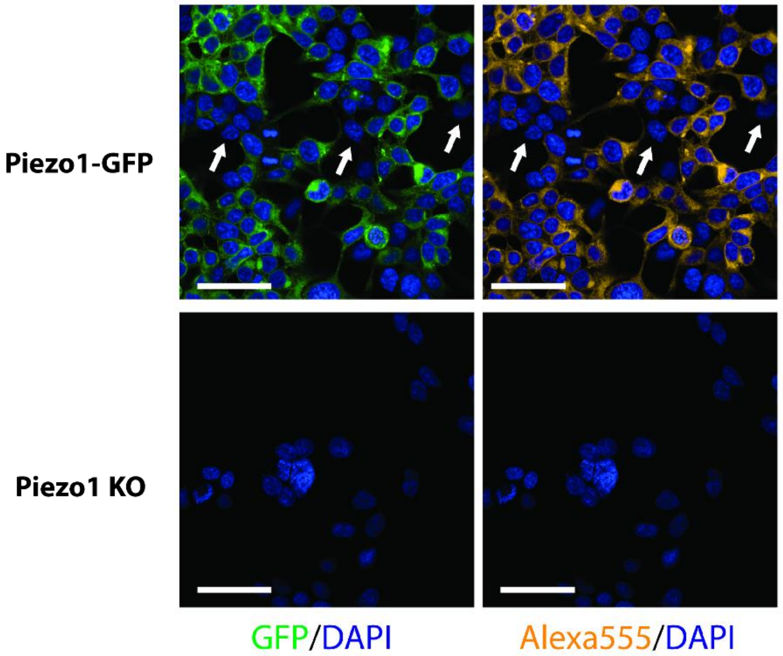

Supplementary Figure 7. Fidelity of mouse monoclonal anti-Piezo1 antibody for immunofluorescence. Immunofluorescence of Piezo1$^{-/-}$ HEK293T cells expressing Piezo1-GFP probed using Novus monoclonal anti-Piezo1 primary antibody and its secondary antibody conjugated with Alexa555 fluorophore. Piezo1$^{-/-}$ HEK293T cells are shown as negative control. DAPI signal represents nuclei. Arrows point out un-transfected cells, which has neither GFP nor Alexa555 signal.

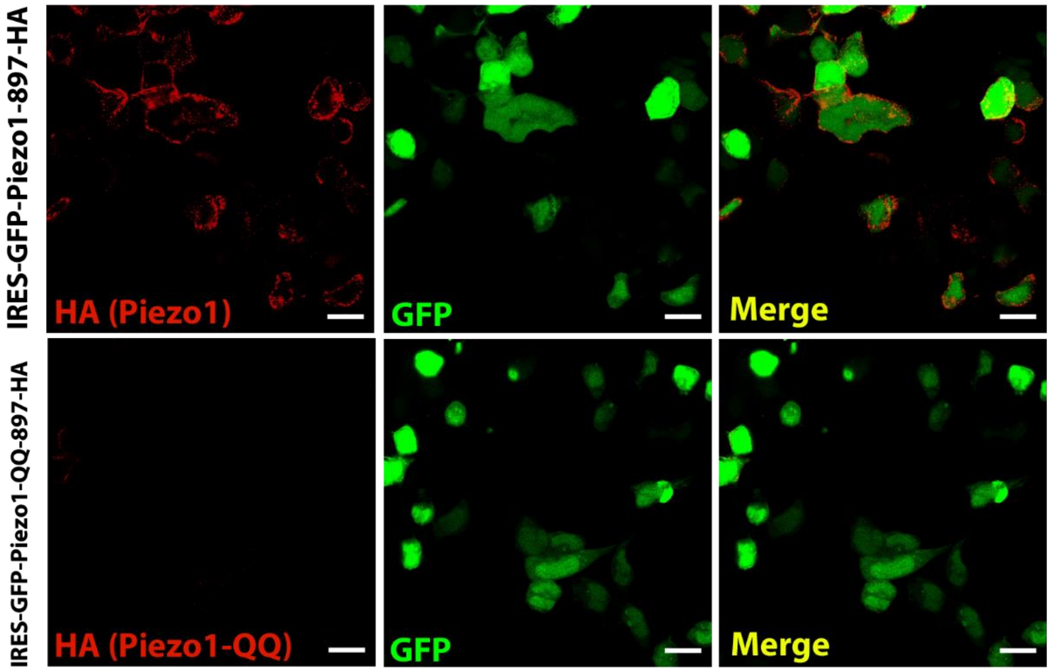

Supplementary Figure 8. Live cell labelling confirms the CapQQ mutant is trafficking defective. Piezo1 $^{-/-}$ HEK293T cells overexpressing Wild-type Piezo1 (Top row) or Piezo1

CapQQ (N2294Q/N2331Q - Bottom row) with an extracellular HA tag (at position 897) were live cell stained with anti-HA antibody and AlexaFluor-555 conjugated goat anti-mouse secondary antibody before 4% PFA fixation. All figures were taken under the same laser power and gain, and presented with the same maximum/minimum intensity. Red channel, signal from HA antibody; green channel, signal from free GFP.

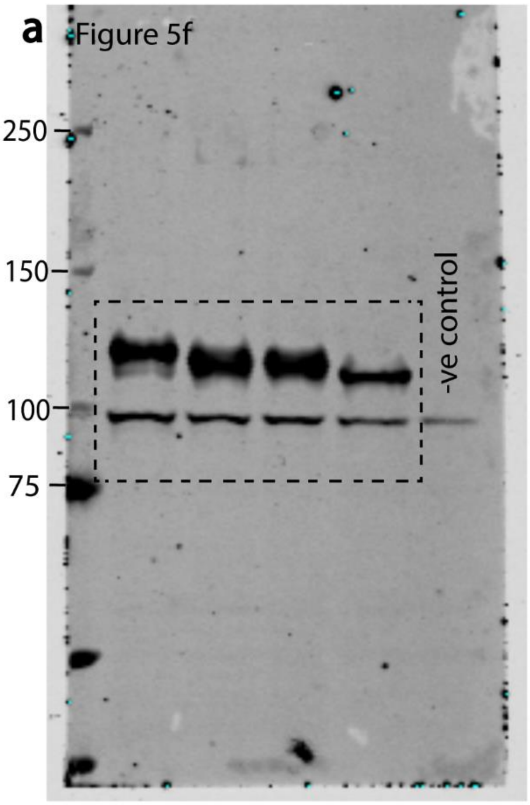

Supplementary Figure 9. Full blot from Figure 5. (a) Blot from Figure 5f. Dashed lines represent cropped areas.

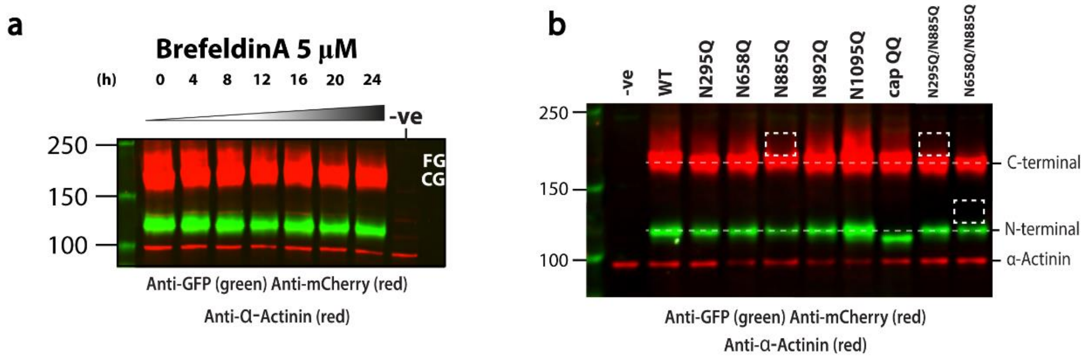

Supplementary Figure 10. Representative western blots of Split Piezo1 protein. (a) Effect of brefeldin A treatment overtime on the human split Piezo1 protein. (b) Representative

western blot showing the comparison of N-terminal Asn to Gln mutations in the N-terminal portion of the human split Piezo1 and the double cap mutant N2294Q and N2331Q (CapQQ).

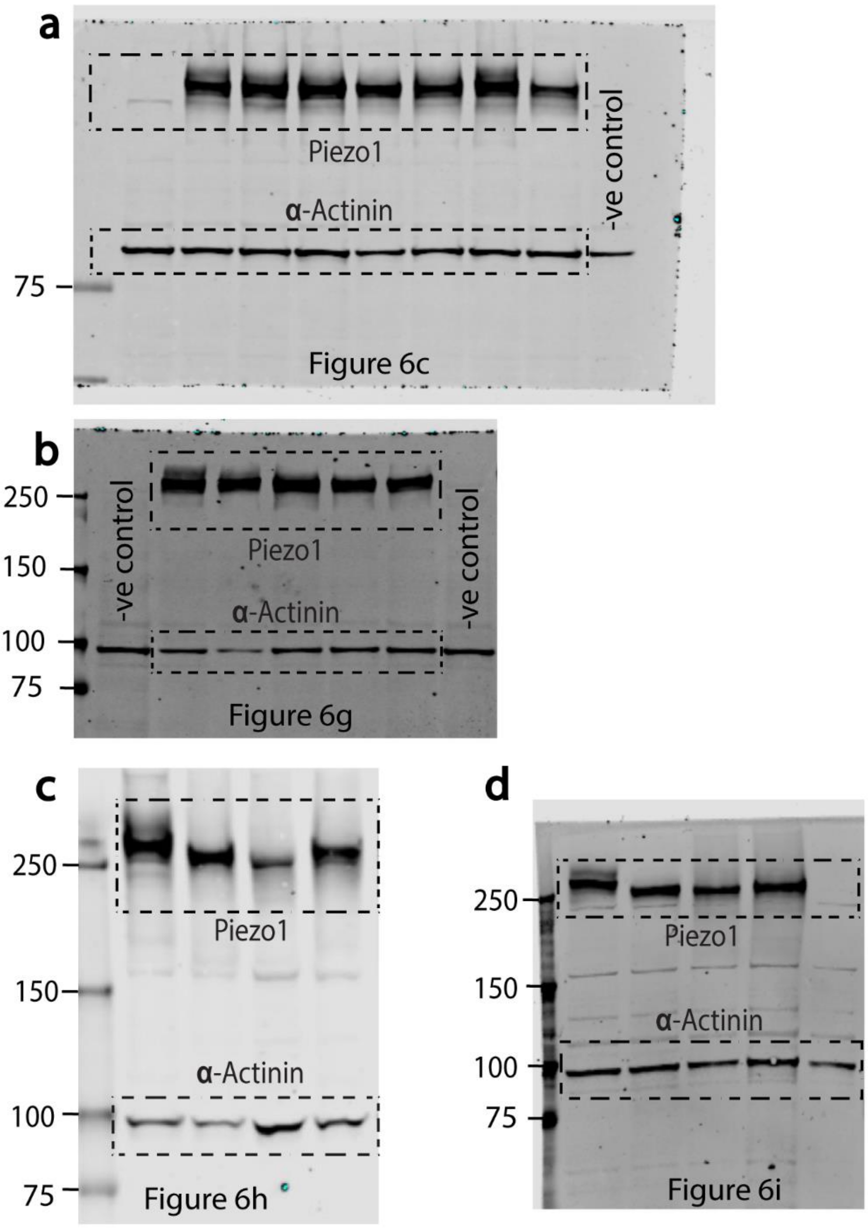

Supplementary Figure 11. Full blots from Figure 6. (a) Blot Figure 6c, (b) Blot Figure 6g, (c) Blot Figure 6h, (d) Blot Figure 6i. Dashed lines represent cropped areas.

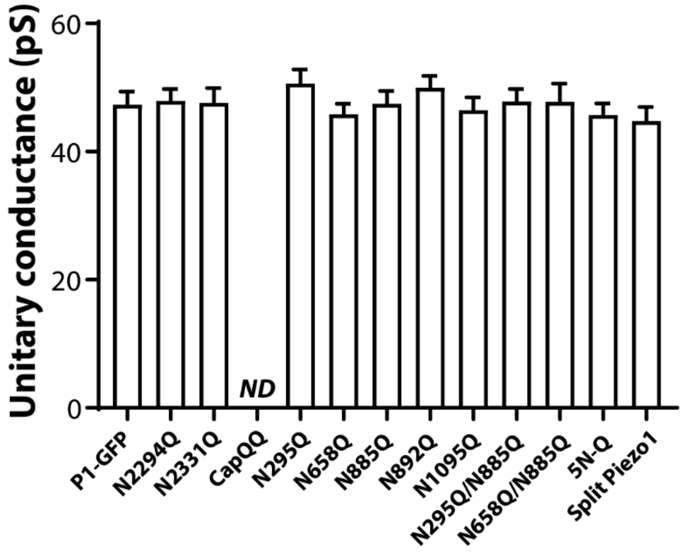

Supplementary Figure 12. Comparison of unitary conductance of glycosylation mutants. Unitary conductance of Piezo1 variants indicated in the presence of high extracellular K^+ to zero membrane potential. Data represents mean ± SEM; n=5-8.

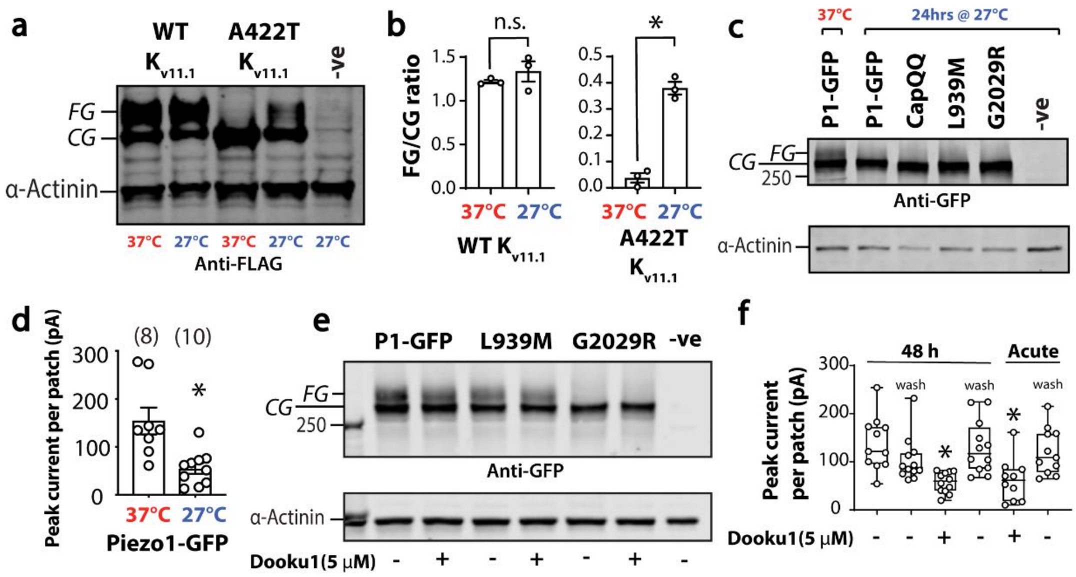

Supplementary Figure 13. Effect of temperature and Piezo1 antagonist dooku-1 on Piezo1 N-glycosylation. (a) As a positive control a representative western blot showing that 27°C treatment for 24 hours does not change the upper FG band of $K_{v11.1}$ WT markedly, but considerably increases the upper FG band while concomitantly decreases the lower CG band of $K_{v11.1}$ A422T mutant which is temperature rescuable. (b) Quantification of upper FG band/lower CG band ratio of samples shown in H. (c) Representative western blot including HEK293T cells incubated at 37°C while expressing Piezo1-GFP as a control, and 27°C for 24 hours for Piezo1-GFP, CapQQ, L939M, and G2029R. (d) Quantification of peak current elicited per patch for Piezo1$^{-/-}$ HEK293T cells expressing P1-GFP incubated at 37°C and 27°C for 24 hours. (e) Representative western blot comparing the effect of 5 μM Dooku1 treatment for 48 h on upper FG bands of P1-GFP, L939M and G2029R. (f) Quantification of peak current elicited per patch showing the effect of treatment with 5 μM Dooku1 on P1-GFP. This includes both 48 h of treatment with Dooku1 compared to washout and acute treatment compared to washout. * $p<0.05$ determined by Kruskal-Wallis test with Dunn's post-hoc test or Mann-Whitney-U test. -ve represents an un-transfected control.

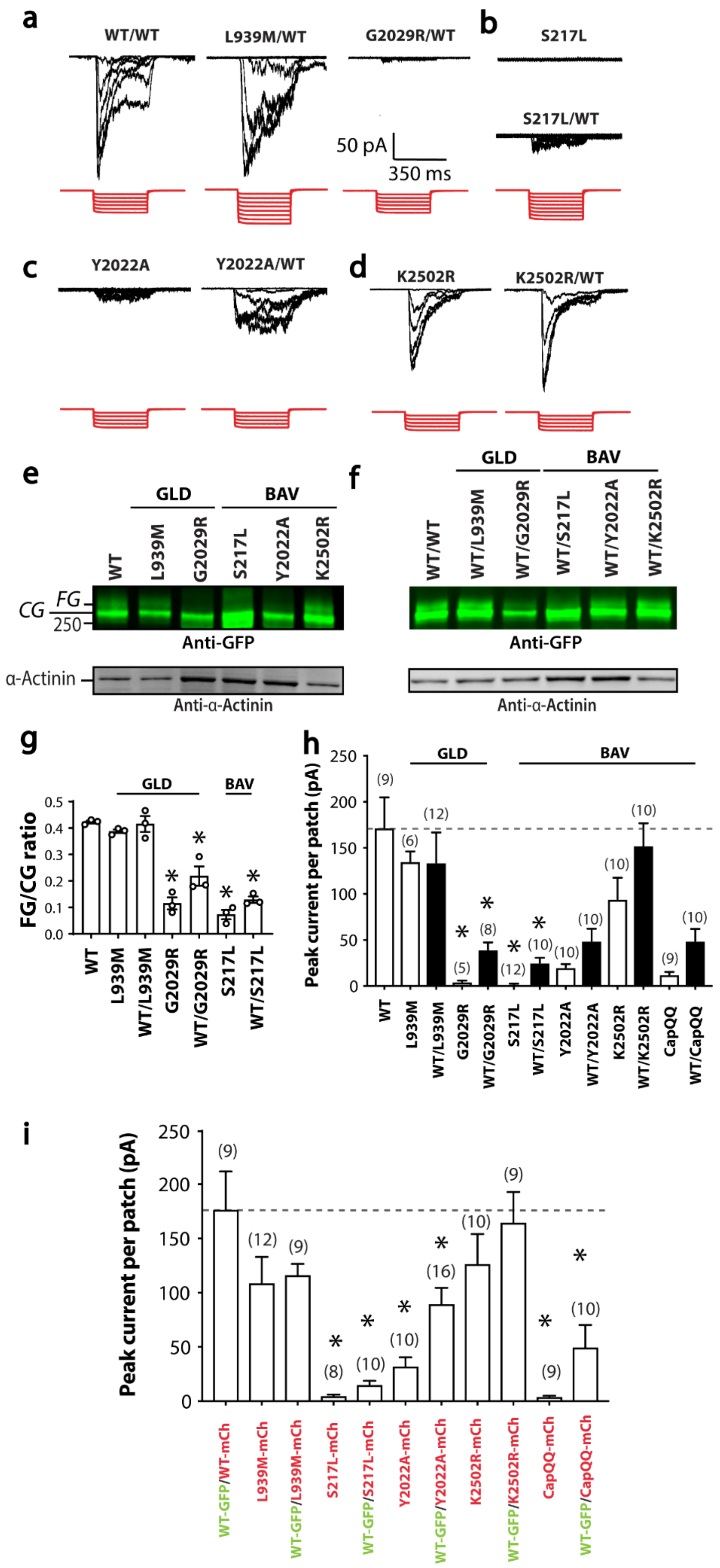
a
WT/WT
L939M/WT
G2029R/WT
50 pA
350 ms
b
S217L
S217L/WT
c
Y2022A
Y2022A/WT
d
K2502R
K2502R/WT
e
GLD
BAV
WT
L939M
G2029R
S217L
Y2022A
K2502R
CG
FG
250
Anti-GFP
α-Actinin
Anti-α-Actinin
f
GLD
BAV
WT/WT
WT/L939M
WT/G2029R
WT/S217L
WT/Y2022A
WT/K2502R
Anti-GFP
Anti-α-Actinin
g
FG/CG ratio
GLD
BAV
WT
L939M
WT/L939M
G2029R
WT/G2029R
S217L
WT/S217L
h
Peak current per patch (pA)
GLD
BAV
WT
L939M
WT/L939M
G2029R
WT/G2029R
S217L
WT/S217L
Y2022A
WT/Y2022A
K2502R
WT/K2502R
CapQQ
WT/CapQQ
i
Peak current per patch (pA)
WT-GFP/WT-mCh
L939M-mCh
WT-GFP/L939M-mCh
S217L-mCh
WT-GFP/S217L-mCh
Y2022A-mCh
WT-GFP/Y2022A-mCh
K2502R-mCh
WT-GFP/K2502R-mCh
CapQQ-mCh
WT-GFP/CapQQ-mCh

Supplementary Figure 14. N-linked glycosylation status of Piezo1 in disease-linked variants co-expressed with WT Piezo1. (a) Electrophysiological recordings of Piezo1$^{-/-}$ HEK293T expressing Piezo1-GFP co-expressed with Piezo1-GFP, L939M, G2029R in the cell-attached configuration in response to negative pressure applied using a high-speed pressure-clamp (red). (b) Electrophysiological recordings of bicuspid aortic valve (BAV) linked mutant S217L alone and co-expressed with Piezo1-GFP. (c) Electrophysiological recordings of Y2022A alone and co-expressed with Piezo1-GFP. (d) Electrophysiological recordings of K2502R, alone and co-expressed with Piezo1-GFP. (e) Representative western blot of Piezo1-GFP, L939M, G2029R, S217L, Y2022A and K2502R. (f) Representative western blot of Piezo1-GFP/Piezo1-GFP, L939M/Piezo1-GFP, G2029R/Piezo1-GFP, S217L/Piezo1-GFP, Y2022A/Piezo1-GFP and K2502R/Piezo1-GFP. (g) Quantification of upper FG band/lower CG band ratio of Piezo1-GFP, L939M, G2029R, S217L, and L939M, G2029R, S217L co-expressed with Piezo1-GFP. (h) Quantification of peak current elicited per patch of Piezo1-GFP, L939M, G2029R, S217L, Y2022A, K2502R, CapQQ and each mutant co-expressed with Piezo1-GFP. (i) Quantification of peak current elicited per patch of Piezo1-GFP/Piezo1-mCherry, L939M-mCherry alone, Piezo1-GFP/L939M-mCherry, S217L-mCherry alone, Piezo1-GFP/S217L-mCherry, Y2022A-mCherry alone, Piezo1-GFP/Y2022A-mCherry, K2502R-mCherry alone, Piezo1-GFP/ K2502R-mCherry, CapQQ-mCherry alone and Piezo1-GFP/CapQQ-mCherry expressed in HEK293T Piezo1$^{-/-}$ cells. * Data represents mean± SEM; $P<0.05$ determined by Kruskal-Wallis test with Dunn's post-hoc test. (GLD – generalized lymphatic dysplasia; BAV – bicuspid aortic valve).

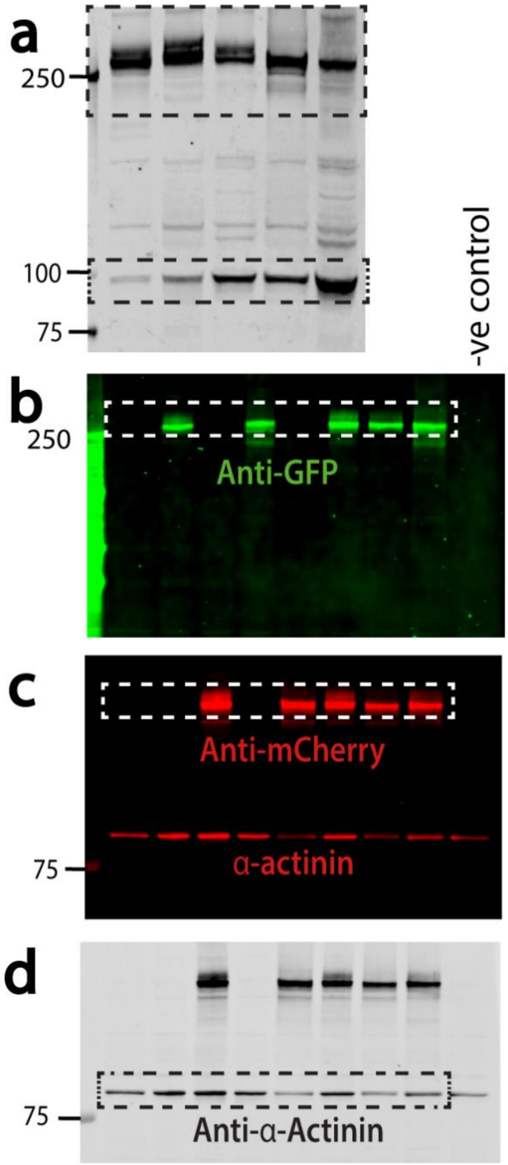

Supplementary Figure 15. Full blots from Figure 8. (a) Blot Figure 8a, (b-d) Blots from Figure 8b. Dashed lines represent cropped areas.

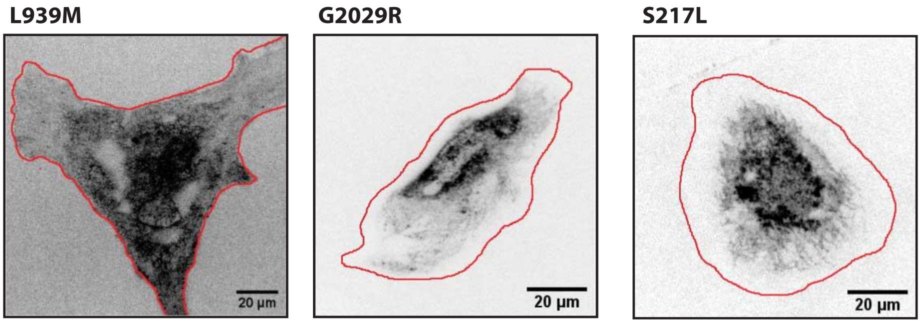

Supplementary Figure 16. Total internal reflection fluorescence (TIRF) microscopy confirms trafficking defects of S217L and G2029R. From left to right shows TIRF images of Piezo1 L939M, S217L and G2029R expressed in human dermal fibroblasts. Note the limited signal of S217L and G2029R indicative of reduced membrane labelling compared to L939M. Red line delineates the cell border identified with epifluorescence.
