# Supplementary material for: Modified N-linked glycosylation status predicts trafficking defective human Piezo1 channel mutations
Source: Commun Biol. 2021 Sep 6;4:1038. doi: 10.1038/s42003-021-02528-w (PMC8421374; doi:10.1038/s42003-021-02528-w)
Supplement: Supplementary file 2 — Description of Additional Supplementary Files [file 42003_2021_2528_MOESM2_ESM.pdf]

Description of Additional Supplementary Files

File name: Supplementary Data 1.

Description: Source data underlying graphs and charts in the main figures.
