# Supplementary material for: Modified N-linked glycosylation status predicts trafficking defective human Piezo1 channel mutations
Source: Commun Biol. 2021 Sep 6;4:1038. doi: 10.1038/s42003-021-02528-w (PMC8421374; doi:10.1038/s42003-021-02528-w)
Supplement: Supplementary file 4 — Reporting Summary [file 42003_2021_2528_MOESM4_ESM.pdf]

nature research

Corresponding author(s): *Double-blind peer review submissions: write DBPR and your manuscript number here instead of author names.*

Last updated by author(s): May 6, 2021

Reporting Summary

Nature Research wishes to improve the reproducibility of the work that we publish. This form provides structure for consistency and transparency in reporting. For further information on Nature Research policies, see our Editorial Policies and the Editorial Policy Checklist.

Statistics

For all statistical analyses, confirm that the following items are present in the figure legend, table legend, main text, or Methods section.

n/a	Confirmed	
☐	☒	The exact sample size (*n*) for each experimental group/condition, given as a discrete number and unit of measurement
☐	☒	A statement on whether measurements were taken from distinct samples or whether the same sample was measured repeatedly
☐	☒	The statistical test(s) used AND whether they are one- or two-sided<br>*Only common tests should be described solely by name; describe more complex techniques in the Methods section.*
☒	☐	A description of all covariates tested
☒	☐	A description of any assumptions or corrections, such as tests of normality and adjustment for multiple comparisons
☐	☒	A full description of the statistical parameters including central tendency (e.g. means) or other basic estimates (e.g. regression coefficient) AND variation (e.g. standard deviation) or associated estimates of uncertainty (e.g. confidence intervals)
☐	☒	For null hypothesis testing, the test statistic (e.g. *F*, *t*, *r*) with confidence intervals, effect sizes, degrees of freedom and *P* value noted<br>*Give P values as exact values whenever suitable.*
☒	☐	For Bayesian analysis, information on the choice of priors and Markov chain Monte Carlo settings
☒	☐	For hierarchical and complex designs, identification of the appropriate level for tests and full reporting of outcomes
☒	☐	Estimates of effect sizes (e.g. Cohen's *d*, Pearson's *r*), indicating how they were calculated

Our web collection on statistics for biologists contains articles on many of the points above.

Software and code

Policy information about availability of computer code

Data collection	N/A
Data analysis	N/A

For manuscripts utilizing custom algorithms or software that are central to the research but not yet described in published literature, software must be made available to editors and reviewers. We strongly encourage code deposition in a community repository (e.g. GitHub). See the Nature Research guidelines for submitting code & software for further information.

Data

Policy information about availability of data

All manuscripts must include a data availability statement. This statement should provide the following information, where applicable:

- Accession codes, unique identifiers, or web links for publicly available datasets
- A list of figures that have associated raw data
- A description of any restrictions on data availability

All data is available from the lead author on request.

Field-specific reporting

Please select the one below that is the best fit for your research. If you are not sure, read the appropriate sections before making your selection.

☒ Life sciences ☐ Behavioural & social sciences ☐ Ecological, evolutionary & environmental sciences

For a reference copy of the document with all sections, see nature.com/documents/nr-reporting-summary-flat.pdf

Life sciences study design

All studies must disclose on these points even when the disclosure is negative.

Sample size	For patch clamping electrophysiology experiment, 7-24 data points were included, and the minimum number of the data points were based on previous studies. For Western blot experiments, at least three blots were analyzed, and this number is chosen based on previous studies.
Data exclusions	No data points were excluded from the analysis of this study.
Replication	Each experiment presented in the paper was repeated at least three times.
Randomization	Data points in this study were not chosen randomly.
Blinding	Investigators were not blinded in the data collection process.

Reporting for specific materials, systems and methods

We require information from authors about some types of materials, experimental systems and methods used in many studies. Here, indicate whether each material, system or method listed is relevant to your study. If you are not sure if a list item applies to your research, read the appropriate section before selecting a response.

Materials & experimental systems

n/a	Involved in the study	
☐	☒	Antibodies
☐	☒	Eukaryotic cell lines
☒	☐	Palaeontology and archaeology
☐	☒	Animals and other organisms
☐	☒	Human research participants
☒	☐	Clinical data
☒	☐	Dual use research of concern

Methods

n/a	Involved in the study	
☒	☐	ChIP-seq
☐	☐	Flow cytometry
☒	☐	MRI-based neuroimaging

Antibodies

Antibodies used	Rabbit monoclonal 159 anti-GFP antibody (Santa Cruz Biotechnology, Dallas, TX, USA)<br>Rat monoclonal anti-mCherry antibody (Clone 16D7, ThermoFisher Scientific,Waltham, MA, USA);<br>Mouse monoclonal anti-Piezo1 antibody (Cat# NBP2-75617, Novus Biologicals, Centennial, CO, USA)<br>Mouse anti-α-actinin antibody (Santa Cruz Biotechnology);<br>Mouse anti-α-tubulin (Clone DM1A, , T9026, Sigma Aldrich, St. Louis, MO, United States);<br>Mouse anti-vinculin antibody (1:200; V9131, Sigma-Aldrich)
Validation	Rabbit monoclonal 159 anti-GFP antibody (Santa Cruz Biotechnology, Dallas, TX, USA), rat monoclonal anti-mCherry antibody (Clone 16D7, ThermoFisher Scientific,Waltham, MA, USA), and mouse monoclonal anti-Piezo1 antibody (Cat# NBP2-75617, Novus Biologicals, Centennial, CO, USA) were validated by us using system null for these proteins, i.e. Piezo1, GFP and mCherry

Eukaryotic cell lines

Policy information about cell lines

Cell line source(s)	HEK293T 140 Piezo1-/- cells, HEK293S GnT1-/- and HEK293 cells (ThermoFisher Scientific, Cat. No. R78007);<br>Human BJ-5ta-hTERT foreskin fibroblasts (UTMB, Galveston, TX, USA)
Authentication	Cell lines were not authenticated.
Mycoplasma contamination	All cell lines were tested negative for mycoplasma contamination.

Commonly misidentified lines (See ICLAC register)	None of the used cell lines is listed in ICLAC database

Animals and other organisms

Policy information about studies involving animals; ARRIVE guidelines recommended for reporting animal research

Laboratory animals	Male and female of Piezo1-Tdtomato mice (The Jackson Laboratory Stock No: 029214) 10 weeks old were used for this study.
Wild animals	No wild animal were used in this study.
Field-collected samples	Mice were housed in light boxes and entrained to a 12:12 light: dark cycle for 1 week before the experiments, were euthanized with carbon dioxide, then lung tissue was harvested for this study.
Ethics oversight	Tissue collection protocols were approved by the Garvan Institute and St. Vincent's Hospital Animal Ethics Committee and were in accordance with the guidelines of the Australian code for the care and use of animals for scientific purposes (8th edition, National Health and Medical Research Council, Canberra, ACT, Australia, 2013) and the Guide for the Care and Use of Laboratory Animals (8th edition, National Research Council, USA, 2011).

Note that full information on the approval of the study protocol must also be provided in the manuscript.

Human research participants

Policy information about studies involving human research participants

Population characteristics	Human participants were an informed-consenting healthy female (age of 26 years) and a male (age of 36 years). All dedicated for no medication treatment or therapy. Blood samples were collected by venipuncture from the cubital fossa.
Recruitment	All participants were randomly chosen informed-consenting normal donors, and there is no self-selection bias or other biases that are likely to influence the result of the study.
Ethics oversight	University of Sydney Human Ethics Committee (signed, approved consent form; Project No. 2012/2882).

Note that full information on the approval of the study protocol must also be provided in the manuscript.

Flow Cytometry

Plots

Confirm that:

- [ ] The axis labels state the marker and fluorochrome used (e.g. CD4-FITC).
- [ ] The axis scales are clearly visible. Include numbers along axes only for bottom left plot of group (a 'group' is an analysis of identical markers).
- [ ] All plots are contour plots with outliers or pseudocolor plots.
- [ ] A numerical value for number of cells or percentage (with statistics) is provided.

Methodology

Sample preparation	N/A
Instrument	N/A
Software	N/A
Cell population abundance	N/A
Gating strategy	N/A

- [ ] Tick this box to confirm that a figure exemplifying the gating strategy is provided in the Supplementary Information.
